# Acute polymyositis associated with hemophagocytic lymphohistiocytosis syndrome

**DOI:** 10.1186/1471-2474-14-S1-A8

**Published:** 2013-02-14

**Authors:** Uma Karjigi, Michael Plant

**Affiliations:** 1 James Cook University Hospital, Middlesbrough, UK

## Background

Hemophagocytic lymphohistiocytosis (HLH) is a life-threatening disease in which an exaggerated but ineffective immune response leads to severe hyper inflammation. It commonly affects infants from birth to 18 months of age, but cases in older children and adults have also been reported. Key players in HLH are activated lymphocytes and histiocytes, they infiltrate all organs and secrete large amounts of cytokines. Cardinal symptoms are prolonged fever, hepatosplenomegaly, cytopenias and hemophagocytosis [1]. The aim of this case presentation is to focus on the unique presentations of HLH syndrome and to be aware of unusual presentations, so that we could diagnose early and treat it appropriately to ensure long term survival.

## Case presentation

19 yr old young boy presented with high fever, rigors & lethargy 5 weeks after been to Spain. Investigations showed neutropenia, lymphopenia & abnormal LFT’s (WCC 1.2, Neut 0.7, ALT 815, ESR 13, CRP<5). Septic screen, HIV, hepatitis screen & Paul bunnel test were negative.

Fever continued despite 9 days of Intravenous antibiotics. CT chest/abdo/pelvis revealed oedematous and thickening of psoas, rectus and paraspinous muscle suggestive of myositis. CK was raised at 7200, subsequent EMG and muscle biopsy confirmed polymyositis.

Reviewed by Haematology team, started on granulocyte colony stimulating factor. Bone marrow biopsy showed increased number of megakaryocytes with evidence of clustering, normocellular marrow with non specific changes. He developed diffuse macular rash, diagnosis of lupus/myositis overlap considered and had 3 doses of pulse IV Methyl prednisolone, then switch to oral prednisolone 60 mg.

He developed shortness of breath due to bilateral pleural effusion leading to hypoxia requiring chest drain. Autoantibody screen including Jo 1, Mi-2, Ku & Pl-7, Ds DNA, anticardiolipin antibody were negative and complements came back as normal. Ferritin was high at 10,000 and continued to have swinging fever, neutropenia and abnormal LFT’s.

On review of clinical presentation and investigations we suspected HLH syndrome and referred back to Haematology. Repeat bone marrow biopsies confirmed heamophagocytosis. He fulfilled criteria for diagnosis of Haemophagocytic lymphohistiocytosis (HLH) and continued with high dose oral steroids & started on Cyclosporine as steroid sparing agent. His blood counts and muscle weakness improved gradually over next 3 months.

## Discussion

Haemophagocytic syndrome can be primary due to mutations in different genes or secondary in association with infections, autoimmune or malignant disorders [2]. A series of adult patients with HLH and a variety of autoimmune diseases including lupus erythematosus, rheumatoid arthritis, Still's disease, polyarteritis nodosa, mixed connective tissue disease, pulmonary sarcoidosis, systemic sclerosis, and Sjogren's syndrome have been reported. However this is the first case reported of HLH syndrome presenting as Polymyositis.

5 out of 9 following criteria should be present to diagnose HLH syndrome [3].

1. Fever

2. Splenomegaly

3. Cytopenias involving two or more cell lines

4. Hypertriglyceridemia or hypofibrinogenemia

5. Hemophagocytosis on bone marrow

6. Hepatitis

7. Low or absent natural killer activity

8. Serum ferritin level > 500 micrograms/l

9. Soluble CD25 > 2400 U/ml

There are published observational data suggest that the TNF inhibitor etanercept is potentially useful for obtaining remission in children not responding to steroids and cyclosporin A [4].
